# Adverse events for biologics in patients with CRSwNP: A meta‐analysis

**DOI:** 10.1002/clt2.12169

**Published:** 2022-06-15

**Authors:** Yang Shen, Xia Ke, Suling Hong, Yucheng Yang

**Affiliations:** ^1^ The First Affiliated Hospital of Chongqing Medical University Chongqing People’s Republic of China

**Keywords:** adverse event, biologics, chronic rhinosinusitis with nasal polyposis, meta‐analysis

1

Chronic rhinosinusitis with nasal polyposis (CRSwNP) is a common and complex inflammatory disorder in the upper airway. Type 2 inflammation affects the severity of CRSwNP and recurrence of polyps, determining the pharmacotherapy and extent of surgery, also associated with asthma comorbidity.^1^ Over the past decade, biologics targeting the biomarkers of type 2 immune reaction ‐‐‐‐ IL‐4, IL‐5, and IgE, have been applied to CRSwNP in phase 2 or 3 clinical trials and emerged as an effective treatment option. However, until now, the safety of these biologics has not been systematically analyzed. Thus, this study aimed to observe the incidence of adverse events (AEs) following the use of biologics, to estimate the safety of biologics in CRSwNP.

A systematic search of PubMed, Medline, and Web of Science from January 2005 to May 2021 using the search terms “monoclonal antibody”, “dupilumab”, “mepolizumab” and “omalizumab” and ‘‘nasal polyposis’’ was conducted. Double‐blind, randomized, placebo‐controlled (RCT) studies with biologics‐treated versus placebo‐treated patients with CRSwNP were included. Eligible patients were aged 18 years or older with bilateral nasal polyps and symptoms of chronic rhinosinusitis despite intranasal corticosteroid therapy before randomization. Patients were required to have a bilateral endoscopic nasal polyp score of at least 5, with a minimum score of 2 for each nostril, and exhibit at least two of the following symptoms: nasal congestion or obstruction and either loss of smell or nasal discharge. Exclusion criteria included the requirement for continuous high‐dose oral corticosteroids, treatment with other biologics in the past 12 months, or asthma exacerbations requiring hospitalization within 4 weeks of screening. Heterogeneity was calculated using the I^2^ statistic and the chi‐squared (*χ* ^2^) test. A p*χ* ^2^ value < 0.1 was considered as a significant heterogeneity. The I^2^ values of 25%, 50%, and 75% were considered to indicate low, medium, and high heterogeneity, respectively. A fixed‐effects model was used to perform the meta‐analysis if I^2^ was <0.5; random‐effects were used for analyses with high heterogeneity (*I* ^2^ > 50%).

After screening and eligibility assessment, we identified a total of 8 clinical trials that were represented in the data set.^2^, ^3^, ^4^, ^5^, ^6^, ^7^ These studies focused on three medications: dupilumab (anti‐IL4), mepolizumab (anti‐IL5), and omalizumab (anti‐IgE). Outcome measures included headache, asthma exacerbation, nasopharyngitis, epistaxis, worsening nasal polyps, injection‐site reaction, and the common cold. To explore the influence of different medicine, in subgroup analysis, we categorized the regimens by class as treatment with omalizumab (3 cohorts), dupilumab (3 cohorts), mepolizumab (2 cohorts).

The overall quality assessment for the included 8 RCTs indicated a low risk of bias. Eight RCTs with 1205 patients were analyzed. Our analysis showed that the risk of asthma exacerbation and worsening nasal polyps was significantly lower in patients treated with monoclonal antibody [RR 0.33, 95% CI 0.19–0.56, *P* < 0.0001, Figure 1A; RR 0.28, 95% CI 0.16–0.49, *p* < 0.00001, Figure 1H]. In the subgroup analysis, both patients who were treated with dupilumab or omalizumab were significantly less likely to experience asthma exacerbation than those treated with placebo]RR 0.27, 95% CI 0.13–0.57; OR 0.31, 95% CI 0.12–0.78, Figure 1B]; whereas the decreased incidence in patients who received mepolizumab failed to reach statistical significance (*P* = 0.79) (RR 0.82, 95% CI 0.18–3.66). The odds of experiencing headache, nasopharyngitis, epistaxis, injection‐site reaction, and common cold were not significantly different between patients treated with monoclonal antibody and placebo; no fatal AE has been reported (Figure 1,C,E,G,I,J).

**FIGURE 1:**
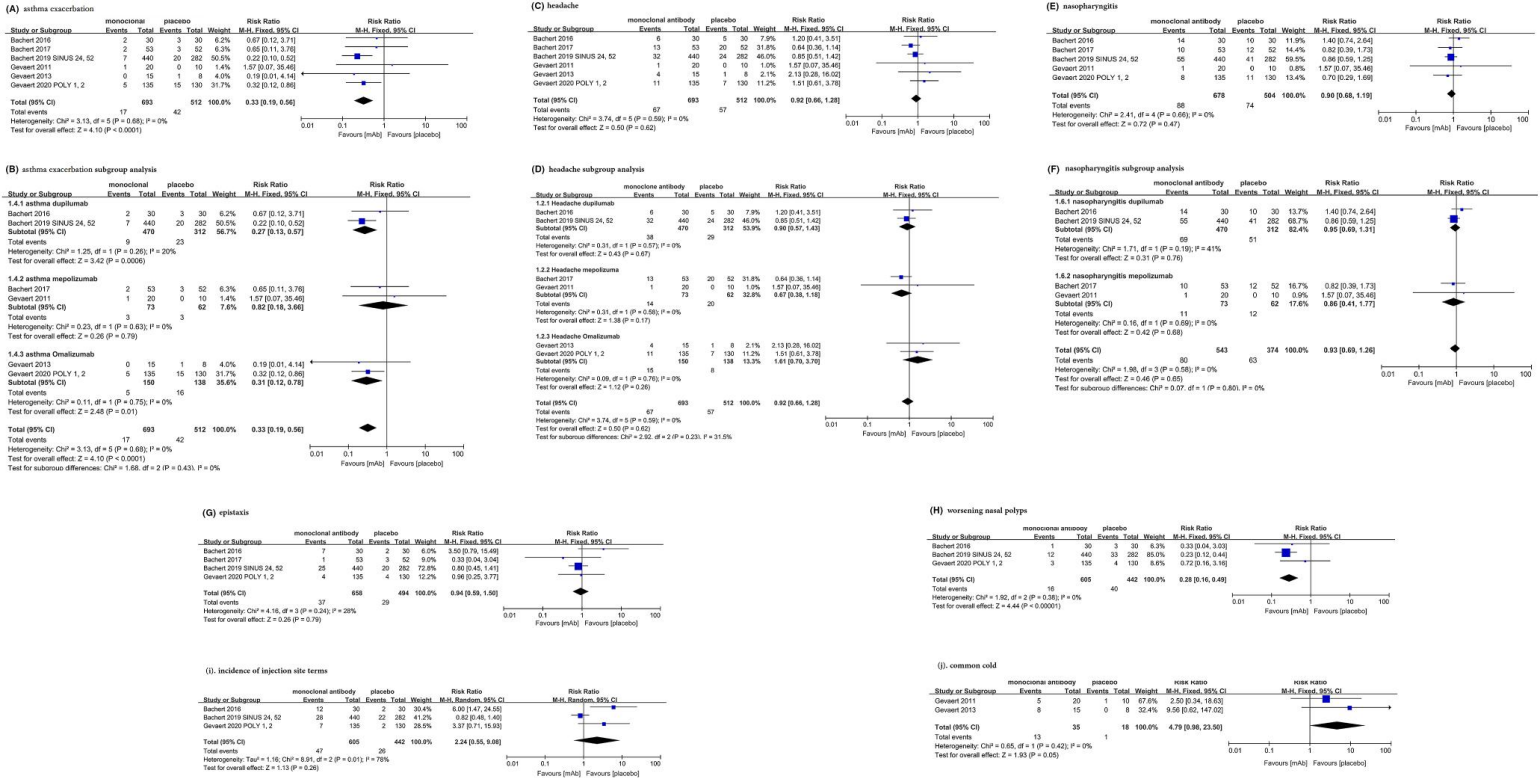
Forest plot of the RCTs comparing rates of patients with asthma exacerbation (A, B), headache (C, D), nasopharyngitis (E, F), epistaxis (G), worsening nasal polyps (H), injection (I) and common cold (J)

In this study, we found that monoclonal antibodies reduced the risk of asthma exacerbation and nasal polyps in CRSwNP. This result could ascribe to the type2 inflammation in nasal polyposis, which is correlated with asthma comorbidity. Type 2 immune response leads to the local production and secretion of IgE, IL‐4, IL‐5, and IL‐13 in the mucosa. These cytokines play a prominent role in the type 2 pathway and contribute to the local persistent inflammation, finally participating in the pathogenesis of nasal polyp formation. Omalizumab, Mepolizumab, and Dupilumab, all these biologics inhibited the functions of type 2 cytokines, then prevented the process of local type 2 inflammation, thus reducing the risk of asthma exacerbation and worsening nasal polyps.

In the past, there were some reports about the AEs associated with monoclonal antibody therapy. Benralizumab, mepolizumab and reslizumab slightly increased drug AE and drug‐related serious AE in severe eosinophilic asthma.^8^ In 362 patients with moderate persistent asthma, who were treated with mepolizumab, nine serious adverse events were reported and two patients suffered from asthma exacerbation.^9^ Samira Jeimy, et al reported a case who developed amyopathic dermatomyositis associated with omalizumab therapy for steroid‐refractory severe asthma.^10^ Besides, Dupilumab‐associated arthralgia and ophthalmic complications (e.g., dry eyes, conjunctivitis, blepharitis, keratitis, and ocular pruritus) were respectively reported in aspirin‐exacerbated respiratory disease and allergic diseases^11^ ^,^ ^12^.

Our results showed that AEs (e.g., headache, asthma exacerbation, nasopharyngitis, epistaxis, worsening nasal polyps, injection‐site reaction, and the common cold)occurred with a similar incidence in both mAb and placebo groups, which indicated the acceptable safety and tolerability of biologics in NPs. Meanwhile, we speculate that CRSwNP patients with other diseases are more likely to suffer from AEs, such as CRSwNP comorbid with asthma. The included research demonstrated high quality and good homogeneity. All these studies were placebo‐controlled RCTs and quality assessment showed a low risk of bias. Also, there was a low heterogeneity in most outcomes across the individual studies. All these strengthen the reliability and credibility of our analysis.

Interestingly, in the pooled data, we observed that there was a slightly increased rate of injection‐site reaction in the monoclonal group. However, the increase failed to reach statistical significance. Will the monoclonal antibody aggravate the erythema at the injection site? It is still uncertain. Meanwhile, in two trials (5, 6), the observed incidence of the common cold was greater in patients who received monoclonal antibodies, although the increase didn't reach significance (*P* = 0.05). Is there any influence of biologics on the immune response to bacteria and viruses? All these questions require more observations.

To conclude, biologics were well tolerated in the application of CRSwNP and decreased the risk of asthma exacerbation and worsening nasal polyps. From a safety perspective, biologics provide a promising innovative option for the treatment of CRSwNP. Further investigations are still needed to confirm our findings and evaluate the longer‐term safety and efficacy of biologics in the use of CRSwNP.

## AUTHOR CONTRIBUTIONS

**Yang Shen:** Writing ‐ original draft; Equal, Writing ‐ review & editing; Equal. **Xia Ke:** Data curation; Equal, Methodology; Equal. **Suling Hong:** Data curation; Equal, Formal analysis; Equal. **Yucheng Yang:** Funding acquisition; Equal, Writing ‐ review & editing; Equal.

## CONFLICT OF INTEREST

The authors declare that no financial or other conflicts of interest exist regarding the content of the article.

## FUNDING INFORMATION

National Natural Science Foundation of China, Grant/Award Number: 81970864; Chongqing Talents Project, Grant/Award Number: cstc2021ycjh‐bgzxm0080
